# Exogenous NAD^+^ decreases oxidative stress and protects H_2_O_2_-treated RPE cells against necrotic death through the up-regulation of autophagy

**DOI:** 10.1038/srep26322

**Published:** 2016-05-31

**Authors:** Ying Zhu, Ke-ke Zhao, Yao Tong, Ya-li Zhou, Yi-xiao Wang, Pei-quan Zhao, Zhao-yang Wang

**Affiliations:** 1 Department of Ophthalmology, Xinhua Hospital, Shanghai Jiaotong University Scool of Medicine, Shanghai, China; 2 Department of Ophthalmology, Shanghai Children’s Medical Center, Shanghai Jiaotong University School of Medicine, Shanghai, China

## Abstract

Increased oxidative stress, which can lead to the retinal pigment epithelium (RPE) cell death by inducing ATP depletion and DNA repair, is believed to be a prominent pathology in age-related macular degeneration (AMD). In the present study, we showed that and 0.1 mM nicotinamide adenine dinucleotide (NAD^+^) administration significantly blocked RPE cell death induced by 300 μM H_2_O_2_. Further investigation showed that H_2_O_2_ resulted in increased intracellular ROS level, activation of PARP-1 and subsequently necrotic death of RPE cells. Exogenous NAD^+^ administration significantly decreased intracellular and intranuclear ROS levels in H_2_O_2_-treated RPE cells. In addition, NAD^+^ administration to H_2_O_2_-treated RPE cells inhibited the activation of PARP-1 and protected the RPE cells against necrotic death. Moreover, exogenous NAD^+^ administration up-regulated autophagy in the H_2_O_2_-treated RPE cells. Inhibition of autophagy by LY294002 blocked the decrease of intracellular and intranuclear ROS level. Besides, inhibition of autophagy by LY294002 abolished the protection of exogenous NAD^+^ against H_2_O_2_-induced cell necrotic death. Taken together, our findings indicate that that exogenous NAD^+^ administration suppresses H_2_O_2_-induced oxidative stress and protects RPE cells against PARP-1 mediated necrotic death through the up-regulation of autophagy. The results suggest that exogenous NAD^+^ administration might be potential value for the treatment of AMD.

Age-related macular degeneration (AMD), a progressive degenerative eye condition, is a leading cause of irreversible severe visual loss in the elderly^1^,^2^. Its prevalence, incidence, and progression of all forms of AMD are estimated to rise due to increase of ageing population worldwide^3^,^4^,^5^. AMD may progress from the early form to the intermediate form and then to the advanced form, where two subtypes exist: the nonneovascular (dry) type (also known as geographic atrophy AMD) and the neovascular (wet) type (also known as exudative AMD)^6^. Loss of vision from this disease is mostly due to the development of neovascular AMD or geographic atrophy (GA). In geographic atrophy AMD, retinal pigment epithelium (RPE) and photoreceptors in the macular area gradually degenerate. Extracellular deposits, called drusen, accumulate between the RPE and Bruch’s membrane, which finally lead to central visual loss. Different from geographic atrophy AMD, exudative AMD is characterized by proliferation of choroideal neovascularization^7^. So, clinically, anti-angiogenesis therapies have been proposed an effective strategy for the treatment of exudative AMD. However, these therapies are too expensive to be available to all patients in many countries^8^,^9^. While, for geographic atrophy AMD, there is still no effective treatment. Thus, the needs for effective and economic therapies on AMD remain urgent.

The progressive degeneration and death of the RPE cells is thought as the initial pathology of AMD^10^. Oxidative stress is considered as one of the major pathological factors involved in the RPE cell death in AMD pathogenesis. It reportedly induced mitochondrial DNA damage and eventually leaded to RPE cell apoptotic death or necrotic death^11^,^12^,^13^. Thus, oxidative stress based therapies are becoming a new strategy for AMD treatment.

Nicotinamide adenine dinucleotide (NAD^+^), a ubiquitous pyridine nucleotide, plays essential roles in many cellular processes such as cellular metabolism, ATP production and DNA repair^14^,^15^. NAD^+^ reportedly attenuates oxidative DNA damages in primary rat cortical neurons^16^. In addition, exogenous NAD^+^ supplementation has been shown to be effective in protecting cardiac myoblasts and neuron against death induced by oxidative stress^17^,^18^. Accordingly, NAD^+^ has been proposed to be a novel and inexpensive cytoprotective agent in the treatment of oxidative stress related-disease. Hence, we hypothesized that exogenous NAD^+^ administration might protect RPE cells from oxidative stress-induced death. However, the effect of NAD^+^ on oxidative stress-induced RPE cell death remains unknown.

In the present study, we examined the effect of exogenous NAD^+^ administration on RPE cell death induced by H_2_O_2_. The results demonstrated that exogenous NAD^+^ administration significantly improved H_2_O_2_ treated-RPE cell viability. In addition, exogenous NAD^+^ administration significantly decreased intracellular and intranuclear ROS level in H_2_O_2_ treated-RPE cell. Furthermore, exogenous NAD^+^ administration inhibited the activation of PARP-1 and protected against RPE cell necrotic death induced by H_2_O_2_ via up-regulating autophagy. The results provide a molecular basis for application of exogenous NAD^+^ administration on the treatment of AMD.

## Materials and Methods

### Cell culture and treatments

Human retina pigment epithelium cells (RPE) were cultured in DMEM/F-12 medium (Gibco, Grand Island, NY, USA) supplemented with 10% heat-inactivated fetal bovine serum (FBS, Gibco, Grand Island, NY, USA) and incubated in a humidified atmosphere with 5% CO_2_ at 37 °C. Solutions of H_2_O_2_ (Sigma, St. Louis, MO, USA), NAD^+^ (Sigma, St. Louis, MO, USA) and LY294002 (Sigma, St. Louis, MO, USA) were freshly prepared in growth culture medium before adding to the cell culture. The RPE cells were incubated at 37 °C for 24 hours after adding different concentration of H_2_O_2_, NAD^+^ or LY294002.

### Cell Counting Kit-8 (CCK-8) Assay

For CCK-8 assay, cells were seeded in 96-well plate at a density of 5 × 10^3^ cells/well with or without 300 μM H_2_O_2_ or different concentration of NAD^+^ and incubated at 37 °C for 24 hours. 10 μL of CCK8 regent (Dojindo Laboratories, Tokyo, Japan) was added to measure cell proliferation by following the manufacturer’s instructions.

### RNA Extraction and Reverse Transcription

For RNA extraction, total RNA was extracted from the RPE cells using Trizol reagent (Invitrogen, Carlsbad, CA, USA) following the supplier’s instructions. One microgram of total RNA was reverse transcribed using the reverse transcription system (Promega, Madison, WI, USA) with oligo (dT) primers.

### Quantitative PCR (qPCR)

SYBR Green (Toyobo, Osaka, Japan) stained quantitative PCR were carried out for analyzing the mRNA expressions of seven enzymes involved in NAD^+^ biosynthesis (QPRT, NMNAT1, NMNAT2, NMNAT3, NMRK1, NMRK2, NAPRT) and GAPDH (as an internal control). The following primers were used: for QPRT: 5′-CTGGTGGAGAAGTATGGGCT-3′ and 5′-AGGTTGTCCAGGGTGATGC-3′; NMNAT2: 5′-CGGTGATGCGGTATGAAGAG-3′ and 5′-ATTCGGTCTGTGTCGGCTGCAT-3′; NMNAT3: 5′- TGGATGGAGACAGTGAAGGTG-3′ and 5′-CACGCACACCAAGCCAAACT-3′; NMRK1: 5′-GGATGGAAAGCGCAAGACAC-3′ and 5′-ATGGCCATCAAAGTATCCCG-3′; NMRK2: 5′-CAGCCCCAAGACCAAATAGCA-3′ and 5′-CACTCTTCATACGGGACGGT-3; NAPRT: 5′-CTCAGGAGATCCGCAAGGTC-3′ and 5′-CCACCAGCTTATAGACGCCA-3′; NMNAT1: 5′-GTGATCTCCGGTAGCACTCG-3′and 5′-ACTGTGTACCTTCCTGTTCCA-3′; for GAPDH: 5′-AAATGGGGTGATGCTGGTGCT-3′ and 5′-AGCCCTTCCACGATGCCAAA -3′; mRNA expressions of targeted gene were normalized to GAPDH mRNA expression, and the relative amounts of all mRNAs were calculated using the comparative Ct method.

### Measurement of NAD^+^ levels and NAD^+^/NADH ratios

NAD^+^ and NADH levels were measured with NAD/NADH quantitation colorimetric kit (Biovison, Milpitas, CA, USA) according to the manufacturer’s instructions.

### Electron microscopy (EM)

RPE cells were fixed overnight in a mixture of cold 2.5% glutaraldehyde in 0.1  M phosphate buffer (pH7.2) and 2% paraformaldehyde in 0.1 M phosphate or cacodylate buffer (pH 7.2) and embedded with epoxy resin. The epoxy resin-mixed samples were loaded into capsules and polymerized at 38 °C for 12 hours and 60 °C for 48 hours. Thin sections were sliced on an ultramicrotome (RMC MT-XL; RMC Products, Tucson, AZ, USA) and collected on a copper grid. Appropriate areas for thin sectioning were cut at 65 nm and stained with saturated 4% uranyl acetate and 4% lead citrate before examination on a transmission electron microscope (JEM-1400; JEOL, Tokyo, Japan) at 80 kV.

### Western Blot

For Western blot, cells were harvested and centrifuged at 800g for 5 minutes to pellet the cell debris. Cells were lysed in RIPA buffer containing proteinase and phosphatase inhibitors. Protein concentrations of the lysates were measured with a BCA protein assay kit (Pierce, Rockford, IL, USA). The lysates were boiled in 4 × sodium dodecyl sulfate loading buffer. 30 μg protein samples were analyzed by SDS-polyacrylamide gel electrophoresis. Then, proteins were transferred to polyvinylidene fluoride membranes (Millipore, Bedford, MA, USA). The blots were blocked for 1 hour in non-fat milk in tris-buffered saline (TBS) and incubated in primary anti-body overnight at 4 °C. The blots were then rinsed thoroughly with TBS and incubated in a 1:1000 dilution of horseradish peroxidase conjugated secondary antibody in TBS for 1 hour at room temperature. HRP signals were developed by using enhanced chemiluminescence (ECL) reagent (Pierce, Rockford, IL, USA) and exposure to X-ray film. Different exposure time was used for each membrane to avoid over-exposure of the bands. The image analysis was performed using BioRad image instrument with multi-analyst software. Rabbit anti-LC3A/B (1:500, Cell Signaling Technology, Beverly, MA, USA), rabbit anti-PARP-1 (1:500, Cell Signaling Technology, Beverly, MA, USA), rabbit anti-Caspase 3 (1:500, Cell Signaling Technology, Beverly, MA, USA), mouse anti-GAPDH (1:500, Sigma, St. Louis, MO, USA) were used as primary antibodies and horseradish peroxidase (HRP)-conjugated goat anti-mouse or goat anti-rabbit IgG (1:1000, Kangcheng, Shanghai, China) as secondary antibodies.

### Detection of autophagy

For the detection of autophagy, cells were seeded in 96-well plate at a density of 5 × 10^3^ cells/well with or without 300 μM H_2_O_2_ or 0.1 mM NAD^+^ treatments and incubated at 37 °C for 24 hours. Autophagy was visualized by LC3 staining using autophagy tandem sensor GFP-LC3B kit (Life Technologies, Carlsbad, CA, USA) according to the manufacturer’s instructions and then images were taken using the Operetta high-content imaging system (PerkinElmer, Waltham, MA, USA) and analyzed using Harmony Software (PerkinElmer, Waltham, MA, USA). Each assay was repeated three times.

### Detection of ROS

For the detection of intracellular ROS, cells were seeded in 96-well plate at a density of 5 × 10^3^ cells/well with or without 300 μM H_2_O_2_ or 0.1 mM NAD^+^ and incubated at 37 °C for 24 hours. Intracellular ROS was detected with CellROX® Reagent (Life Technologies, Carlsbad, CA, USA) according to the manufacturer’s instructions and then images were taken using the Operetta high-content imaging system (PerkinElmer, Waltham, MA, USA) and analyzed using Harmony Software (PerkinElmer, Waltham, MA, USA) to determine the ROS levels. Each assay was repeated three times.

### Detection of apoptosis and necrotic death

For the detection of apoptosis and necrotic death, cells were seeded in 96-well plate at a density of 5 × 10^3^ cells/well with or without 300 μM H_2_O_2_ or 0.1 mM NAD^+^ and incubated at 37 °C for 24 hours. Apoptosis and necrotic death was detected with annexin V fluorescence apoptosis detection kit (BD bioscience, Bedford, MA, USA) and then images were taken using the Operetta high-content imaging system (PerkinElmer, Waltham, MA, USA) and analyzed using Harmony Software (PerkinElmer, Waltham, MA, USA) to determine the proportion of apoptotic cells or necrotic cells. Each assay was repeated three times.

### Statistical Analysis

All experiments were repeated at least three times independently. All data were expressed as means ± SEM and statistical analysis was performed using GraphPad Prism5. The data were analyzed by one-way ANOVA with post hoc test or unpaired t test, as appropriate (*p < 0.05, **p < 0.01, and ***p < 0.001).

## Results

### NAD^+^ blocked the H_2_O_2_-induced RPE cell death

**Figure 1:**
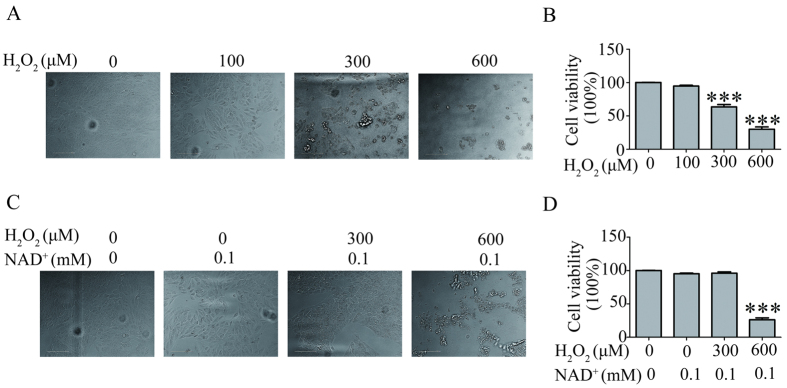
NAD^+^ blocked the H_2_O_2_-induced RPE cell death. (**A**) Representative images show the damage of RPE cells treated without (0), or with different concentration of H_2_O_2_ (100 μM, 300 μM, 600 μM) for 24 hours. (**B**) Cell viability of RPE cells treated without (0), or with different concentration of H_2_O_2_ (100 μM, 300 μM, 600 μM) for 24 hours was assessed by CCK8. (**C**) Representative images show the effect of 0.1 mM NAD^+^ on the RPE cell treated without (0), or with different concentration of H_2_O_2_ (300 μM, 600 μM) for 24 hours. (**D**) The RPE cells treated without (0) or with different concentration of H_2_O_2_ (300 μM, 600 μM) treatment, or without (0) or with 0.1 mM NAD^+^ for 24 hours, cell viability were assessed by CCK8. Data are expressed as mean ± SEM from at least three independent experiments; ***P < 0.001 versus cell viability of RPE cells without any treatment.

Before we identified the effect of NAD^+^ on the H_2_O_2_-induced RPE cell death, we examined the effect of different concentration of H_2_O_2_ on the RPE cells by CCK-8 assay. The result showed that the viability of RPE cell treated with 300 μM and 600 μM H_2_O_2_ for 24 hours significantly reduced by 40% and 80% compared with control cells, respectively. No significant difference was observed between the viability of RPE cells treated with 100 μM H_2_O_2_ for 24 hours and control cells (Fig. 1A,B). Then, we also investigated the effect of different concentration of NAD^+^ on the viability of RPE cells by CCK8 assay to determine the concentration of NAD^+^ we treated on the RPE cells. The results showed that no significant difference was observed in the RPE cells treated without NAD^+^ and in the RPE cells treated with 0.01 mM NAD^+^, and in the RPE cells treated with 0.1 mM NAD^+^. However, the cell viability was significantly decreased in the cultured RPE cells treated with 1 mM or 20 mM NAD^+^ compared that in the RPE cells treated without or with 0.01 mM, or 0.1 mM NAD^+^ (Supplementary Figure S1A,B). Accordingly, we treated H_2_O_2_-induced RPE cell with 0.1 mM NAD^+^. Finally, we investigated the effect of NAD^+^ on the H_2_O_2_-induced RPE cell death by CCK-8 assay. The results showed that viability of RPE cells treated with 300 μM H_2_O_2_ and 0.1 mM NAD^+^ significantly increased compared with that of RPE cells treated with 300 μM H_2_O_2_ alone. However, addition of 0.1 mM NAD^+^ to RPE cells treated with 600 μM H_2_O_2_ had no significant difference in the cell viability compared with RPE cells treated with 600 μM H_2_O_2_ alone (Fig. 1C,D). The results indicate that 0.1 mM NAD^+^ protected RPE cells from death induced by 300 μM H_2_O_2_.

### H_2_O_2_ treatment did not affect NAD^+^ biosynthesis

**Figure 2:**
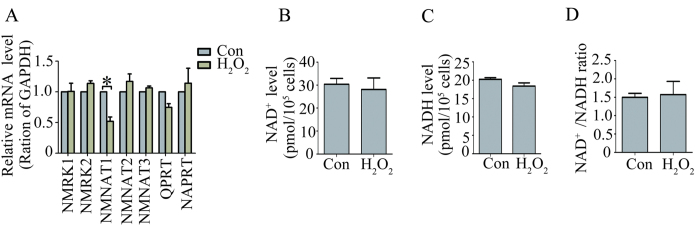
H_2_O_2_ treatment did not affect NAD^+^ biosynthesis. (**A**) mRNA expression levels of seven genes involved in NAD^+^ biosynthesis were measured in cultured RPE cell treated without H_2_O_2_ (Con) or with 300 μM H_2_O_2_ (H_2_O_2_) by quantitative RT-PCR. (**B**) NAD^+^ level measured in the cultured RPE cells treated without H_2_O_2_ (Con) or with 300 μM H_2_O_2_ (H_2_O_2_) for 24 hours. (**C**) NADH level measured in RPE cells treated without H_2_O_2_ (Con) or with 300 μM H_2_O_2_ (H_2_O_2_) for 24 hours. (**D**) Ratio of NAD^+^/NADH in RPE cells treated without H_2_O_2_ (Con) or with 300 μM H_2_O_2_ (H_2_O_2_) for 24 hours. Data are expressed as mean ± SEM from at least three independent experiments; *P < 0.05.

NAD^+^ depletion reportedly causes cell death under cytotoxic stress^19^. Given the protection of exogenous NAD^+^ against the death of RPE cell induced by H_2_O_2_, we supposed that H_2_O_2_ might induce RPE cell death by affecting NAD^+^ production. To test this hypothesis, we first examined the gene expression of seven enzymes involved in NAD^+^ biosynthesis in the RPE cells treated with or without 300 μM H_2_O_2_ using quantitative RT-PCR. The PCR results showed that only the expression of NMNAT1 was significantly decreased in the RPE cells treated with 300 μM H_2_O_2_ compared with that in the RPE cells treated without H_2_O_2_. No significant differences were observed in the expressions of other six enzymes in the RPE cells between H_2_O_2_-treated group and control group (Fig. 2A). Furthermore, we measured NAD^+^ levels and NAD^+^/NADH ratios in the RPE cells treated with or without 300 μM H_2_O_2_. The result showed that no significant differences were observed in the NAD^+^ levels, NADH levels or NAD^+^/NADH ratio in the RPE cells between H_2_O_2_-treated group and control group (Fig. 2B–D). The above results suggest that H_2_O_2_-induced RPE cell death has not been through the inhibition on NAD^+^ biosynthesis or NAD^+^ depletion.

### NAD^+^ reduced intracellular and intranuclear ROS levels in the H_2_O_2_ treated-RPE cells

**Figure 3:**
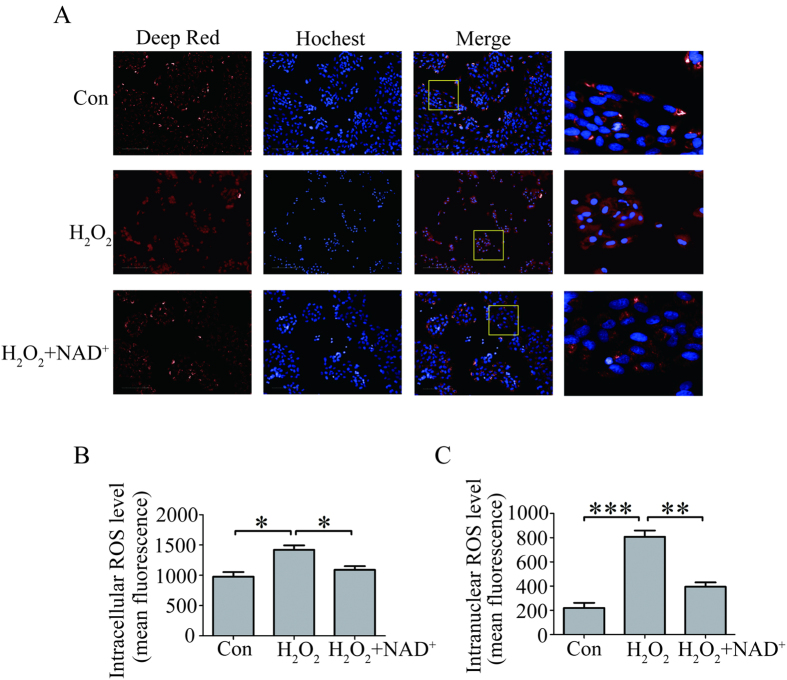
NAD^+^ reduced ROS level in the cultured RPE cells induced by H_2_O_2_. (**A**) Representative high content screening images for the ROS level stained by deep red in the cultured RPE cells treated without H_2_O_2_ (Con) or alone with 300 μM H_2_O_2_ (H_2_O_2_), or with 300 μM H_2_O_2_ and 0.1 mM NAD^+^ (H_2_O_2_^+^ NAD^+^) for 24 hours. Nuclei were labeled with Hochest 33342. Scale bar equals 100 μm. (**B**) Statistical data from (**A**) showing the intracellular ROS level. (**C**) Statistical data from (**A**) showing the intranuclear ROS level. Data are expressed as mean ± SEM from at least three independent experiments; *P < 0.05, **P < 0.01, ***P < 0.001.

Increased oxidative stress is thought as the major cause for RPE cell death. Then, we speculated that the protection of NAD^+^ against H_2_O_2_-induced RPE cell death may associated with the alteration of intracellular oxidative stress. To test this hypothesis, we investigated the effect of NAD^+^ on the alteration of intracellular reactive oxygen species (ROS). The result showed that intracellular ROS significantly increased in the RPE cells treated with 300 μM H_2_O_2_ compared with that in RPE cells without H_2_O_2_ treatment. However, intracellular ROS significantly decreased in the RPE cells treated with 0.1 mM NAD^+^ and 300 μM H_2_O_2_ compared with that in RPE cells treated with 300 μM H_2_O_2_ alone (Fig. 3A,B). Furthermore, we analyzed the intranuclear ROS level in the RPE cells. The result showed that treatment of 300 μM H_2_O_2_ significantly increased intranuclear ROS level in the RPE cells. While, intranuclear ROS level significantly decreased in the RPE cells treated with 0.1 mM NAD^+^ and 300 μM H_2_O_2_ compared with that in RPE cells treated with 300 μM H_2_O_2_ alone (Fig. 3A,C).

### NAD^+^ protected RPE cells from H_2_O_2_-induced necrotic death by inhibiting the activation of PARP-1

**Figure 4:**
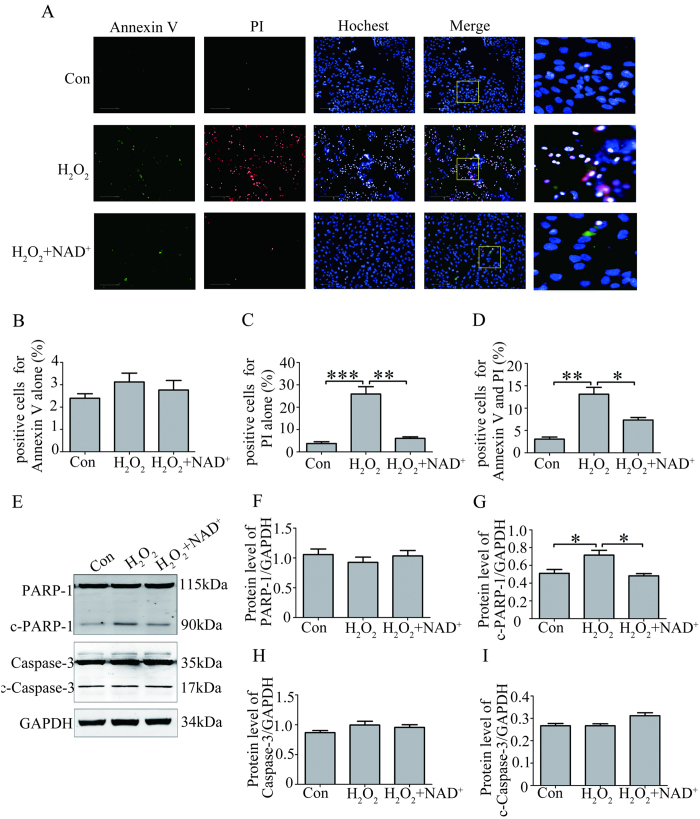
NAD^+^ protected RPE cells against H_2_O_2_-induced necrotic death. (**A**) Representative high content screening images for the Annexin V and PI staining in the cultured RPE cells treated without H_2_O_2_ (Con) or alone with 300 μM H_2_O_2_ (H_2_O_2_), or with 300 μM H_2_O_2_ and 0.1 mM NAD^+^ (H_2_O_2_^+^ NAD^+^) for 24 hours. Nuclei were labeled with Hochest 33342. Scale bar equals 100 μm. (**B**) Statistical data from (**A**) showing the percentage of positive cells for Annexin V staining alone. (**C**) Statistical data from (**A**) showing the percentage of positive cells for PI staining alone. (**D**) Statistical data from (**A**) showing the percentage of positive cells for bothe Annexin V and PI staining. (**E**) Representative western blot images for the expression of PARP-1, cleaved PARP-1(c-PARP-1), Caspase-3 and cleaved Caspase-3 (c-Caspase-3) in the cultured RPE cells treated without H_2_O_2_ (Con) or alone with 300 μM H_2_O_2_ (H_2_O_2_), or with combination of 300 μM H_2_O_2_ and 0.1 mM NAD^+^ (H_2_O_2_^+^ NAD^+^) for 24 hours. (**F**) Statistical data from (**E**) showing the protein ratio of PARP-1/GAPDH. (**G**) Statistical data from (**E**) showing the protein ratio of c-PARP-1/GAPDH. (**H**) Statistical data from (**E**) showing the protein ratio of Caspase-3/GAPDH. (**I**) Statistical data from (**E**) showing the protein ratio of c-Caspase-3/GAPDH. GAPDH as internal control. Data are expressed as mean ± SEM from at least three independent experiments; *P < 0.05, **P < 0.01, ***P < 0.001.

Hydrogen peroxide has been shown to induce apoptosis in RPE cells^12^,^20^,^21^. However, recent study reports that necrotic cell death is a major mechanism for RPE cell death in response to oxidative stress^13^. To determine the effect of NAD^+^ on the two major types of cell death induced by H_2_O_2_, we first performed annexin V/propidium iodide (PI) staining assay detected by high-content screening system (HCS). Cells were judged to be viable if double negative, as early apoptotic if positive for annexin V alone, as necrotic if positive for PI and as necrotic or late apoptotic if double positive. The result showed that no significant difference in the percentage of cells positive for Annexin V was observed in the RPE cells treated with 0.1 mM NAD^+^ and 300 μM H_2_O_2_ compared with that in the RPE cells treated with 300 μM H_2_O_2_ alone, or compared with that in the control RPE cells for 24 hours (Fig. 4A,B). However, the percentage of cells positive for PI showed a significant increase in the RPE cells treated with 300 μM H_2_O_2_ for 24 hours. While, the percentage of cells positive for PI was significantly decreased in the RPE cells treated with 0.1 mM NAD^+^ and 300 μM H_2_O_2_ for 24 hours compared with that in the RPE cells treated with 300 μM H_2_O_2_ alone. However, no significant difference in the percentage of cells positive for PI was observed in the RPE cells treated with 0.1 mM NAD^+^ and 300 μM H_2_O_2_ and in the control RPE cells for 24 hours (Fig. 4A,C). Similarly, the percentage of cells positive for PI and Annexin V was also significantly increased in the RPE cells treated with 300 μM H_2_O_2_ alone compared with that in the RPE cells treated with 0.1 mM NAD^+^ and 300 μM H_2_O_2_ or in the control RPE cells for 24 hours. No significant difference in the percentage of cells positive for PI and Annexin V was observed in the RPE cells treated with 0.1 mM NAD^+^ and 300 μM H_2_O_2_ and in the control RPE cells for 24 hours (Fig. 4A,D).

Poly(ADP-ribose) polymerase-1 (PARP-1) activation is thought as a hallmark of oxidative stress–induced necrotic cell death^22^. To determine the effect of NAD^+^ on the PARP, we investigated the expression of PARP-1 and its active form by western blot. The result showed that the expression of cleaved PARP-1 (active form of PARP-1) significantly increased in RPE cells treated with 300 μM H_2_O_2_ compared with that in the control RPE cells. However, the expression of cleaved PARP-1 significantly decreased in the RPE cells treated with 0.1 mM NAD^+^ and 300 μM H_2_O_2_ compared with that in the RPE cells treated with 300 μM H_2_O_2_ alone. No significant difference was observed in the expression of cleaved PARP-1 between the RPE cells treated with 0.1 mM NAD^+^ and 300 μM H_2_O_2_ and control RPE cells. Besides, no significant difference was observed in the expression of PARP-1 among the three groups (Fig. 4E–G). Furthermore, we investigated the expression of caspase-3, apoptosis-related marker, by the western blot assay. The result showed that no obvious alterations in the expressions of caspase-3 and cleaved caspases-3 were observed among the three groups (Fig. 4E,H,I).

The above results demonstrated that inhibition of NAD^+^ on the oxidative stress–induced activation of PARP-1 protected RPE cells from necrotic death.

### NAD^+^ up-regulated autophagy in the RPE cells treated with H_2_O_2_

**Figure 5:**
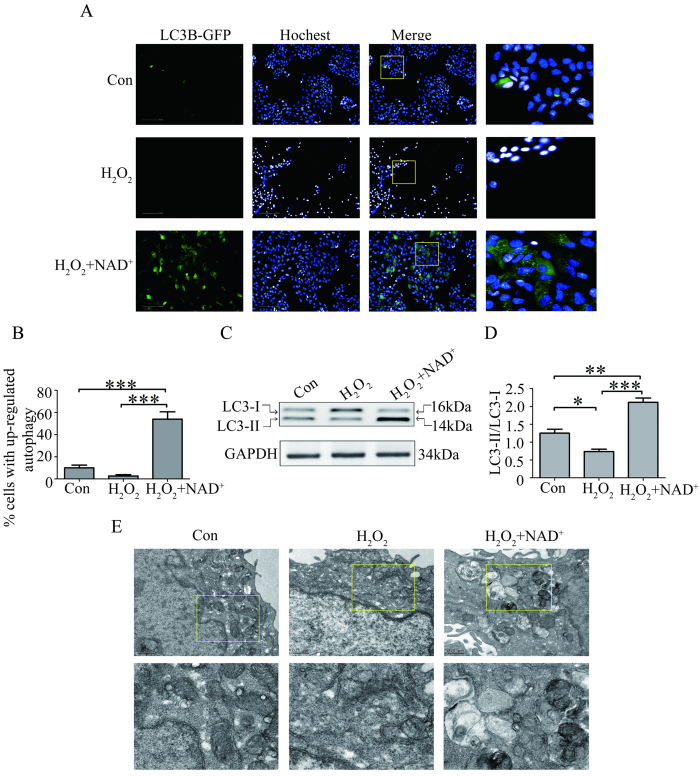
NAD^+^ up-regulated autophagy in the cultured RPE cells treated with H_2_O_2_. (**A**) Representative high content screening images for the autophagosomes in LC3B-GFP-transfected RPE cells treated without H_2_O_2_ (Con) or alone with 300 μM H_2_O_2_ (H_2_O_2_), or with 300 μM H_2_O_2_ and 0.1 mM NAD^+^ (H_2_O_2_^+^ NAD^+^) for 24 hours. Nuclei were labeled with Hochest 33342. Scale bar equals 100 μm. (**B**) Statistical data from (**A**) showing the percentage of cells with up-regulated autophagy by counting the number of GFP-LC3 puncta in each cell. (**C**) Representative western blot images for the expression of LC3B-I and LC3B-II in the RPE cells treated without H_2_O_2_ (Con) or alone with 300 μM H_2_O_2_ (H_2_O_2_), or with 300 μM H_2_O_2_ and 0.1 mM NAD^+^ (H_2_O_2_^+^ NAD^+^) for 24 hours. (**D**) Statistical data from (**C**) showing the protein ratio of LC3B-II/LC3B-I. GAPDH as internal control. (**E**) Representative electron microscope images of autophagosomes in the RPE cells treated without H_2_O_2_ (Con) or alone with 300 μM H_2_O_2_ (H_2_O_2_), or with 300 μM H_2_O_2_ and 0.1 mM NAD^+^ (H_2_O_2_^+^ NAD^+^) for 24 hours.

Autophagy reportedly suppresses necrotic cell death^22^. To identify whether autophagy is involved in the protection of NAD^+^ against H_2_O_2_-induced RPE cells necrotic death, we first transfected RPE cells with autophagy sensor LC3B-GFP to examine the formation of autophagosome by HSC. The result showed that the RPE cells treated with 0.1 mM NAD^+^ and 300 μM H_2_O_2_ showed significant increases in GFP-LC3B autophagic puncta compared with that in RPE cells treated with 300 μM H_2_O_2_ alone and in the control RPE cells (Fig. 5A,B). To confirm the positive effect of autophagy, we had treated RPE cell with rapamycin, a well-known autophagy inducer, which was also used as a positive control for autophagy. The result showed that GFP-LC3B autophagic puncta significantly increased in RPE cells treated with rapamycin (Supplementary Figure S2). Furthermore, we investigated the activation of widely used autophagosome marker LC3B by western blot. The result showed that in H_2_O_2_- and NAD^+^-treated RPE cells, the ratio of LC3B-II/LC3B-I significantly increased compared with that in the RPE cells treated with 300 μM H_2_O_2_ alone and in the control RPE cells (Fig. 5C,D). In addition, we characterized the autophagic response of RPE cells using transmission electron microscopy (TEM). In accordance with the result of HSC, the TEM result showed that number of autophagosome significantly increased in the RPE cells treated with 0.1 mM NAD^+^ and 300 μM H_2_O_2_ compared with that in the RPE cells treated with 300 μM H_2_O_2_ alone and in the control RPE cells (Fig. 5E). These results demonstrated that NAD^+^ treatment enhanced autophagy in the RPE cells exposed to H_2_O_2_.

### Blocking autophagy inhibited the decrease of intracellular and intranuclear ROS level mediated by NAD^+^ in the H_2_O_2_ treated-RPE cells

**Figure 6:**
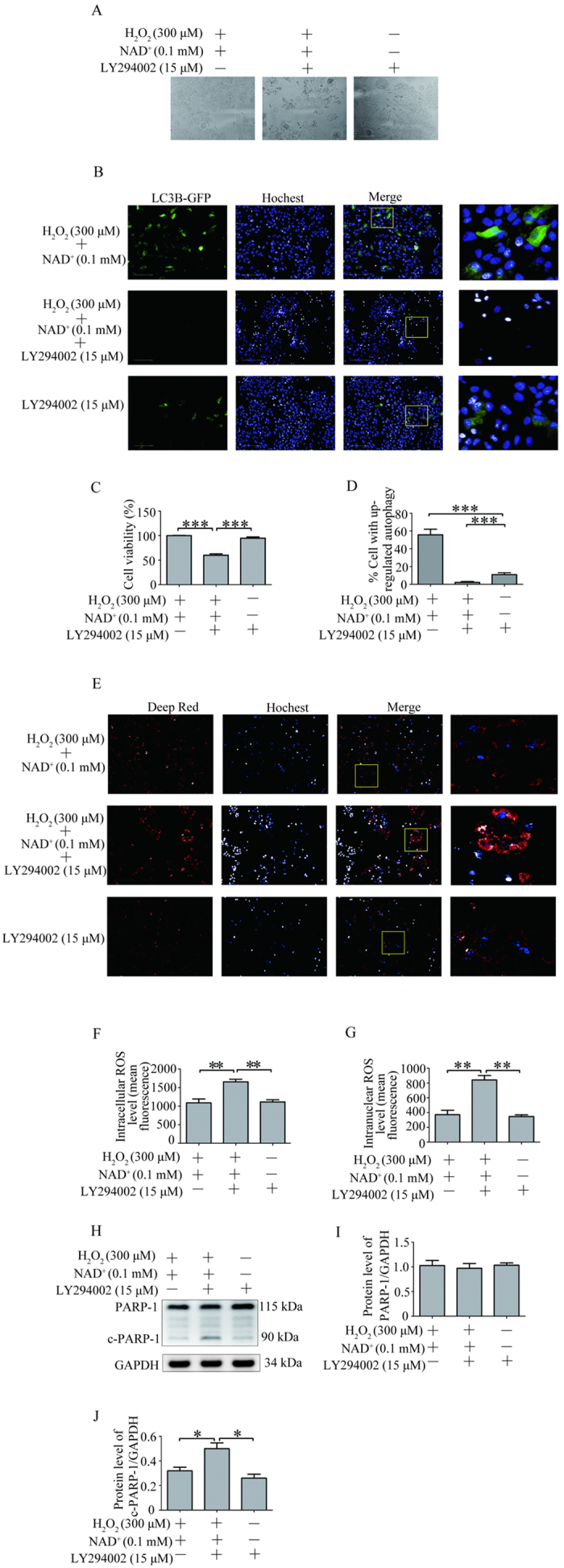
Autophagy inhibitor LY294002 blocked the decrease of intracellular and intranuclear ROS level mediated by NAD^+^ in the H_2_O_2_ treated-RPE cells. (**A**) Representative images show the damage of RPE cells treated with 300 μM H_2_O_2_ and 0.1 mM NAD^+^, or with 300 μM H_2_O_2_, 0.1 mM NAD^+^ and 15 μM LY294002, or with 15 μM LY294002 alone for 24 hours. (**B**) Representative high content screening image for the autophagosomes in LC3B-GFP-transfected RPE cells treated with 300 μM H_2_O_2_ and 0.1 mM NAD^+^, or with 300 μM H_2_O_2_, 0.1 mM NAD^+^ and 15 μM LY294002, or with 15 μM LY294002 alone for 24 hours. Nuclei were labeled with Hochest 33342. Scale bar equals 100 μm. (**C**) Cell viability of RPE cells treated with 300 μM H_2_O_2_ and 0.1 mM NAD^+^, or with 300 μM H_2_O_2_, 0.1 mM NAD^+^ and 15 μM LY294002, or with 15 μM LY294002 alone for 24 hours was assessed by CCK8. (**D**) Statistical data from (**B**) showing the percentage of cells with up-regulated autophagy by counting the number of GFP-LC3 puncta in each cell. (**E**)Representative high content screening image for the ROS level stained by deep red in the cultured RPE cells treated with 300 μM H_2_O_2_ and 0.1 mM NAD^+^, or with 300 μM H_2_O_2_, 0.1 mM NAD^+^ and 15 μM LY294002, or with 15 μM LY294002 alone for 24 hours. Nuclei were labeled with Hochest 33342. Scale bar equals 100 μm. (**F**) Statistical data from (**E**) showing the intracellular ROS level. (**G**) Statistical data from (**E**) showing the intranuclear ROS level. (**H**) Representative western blot images for the expression of PARP-1 and cleaved PARP-1(c-PARP-1) in the cultured RPE cells treated with 300 μM H_2_O_2_ and 0.1 mM NAD^+^, or with 300 μM H_2_O_2_, 0.1 mM NAD^+^ and 15 μM LY294002, or with 15 μM LY294002 alone for 24 hours. (**I**) Statistical data from (**H**) showing the protein ratio of PARP-1/GAPDH. (**J**) Statistical data from (**H**) showing the protein ratio of c-PARP-1/GAPDH. Data are expressed as mean ± SEM from at least three independent experiments; *P < 0.05, **P < 0.01, ***P < 0.001.

Based on the results described above, we speculated that the influence of NAD^+^ on the decrease of ROS might be mediated by its up-regulation on autophagy. To test this hypothesis, we first examined the influence of LY294002, an inhibitor of autophagy, on the viability of REP cells treated with 0.1 mM NAD^+^ and 300 μM H_2_O_2_ by CCK-8 assay. The result showed that the viability of REP cells treated with 300 μM H_2_O_2_, 0.1 mM NAD^+^ and 15 μM LY294002 significantly decreased compared with the viability of REP cells treated with 0.1 mM NAD^+^ and 300 μM H_2_O_2_ and the viability of REP cells treated with 15 μM LY294002 alone (Fig. 6A,C). Furthermore, we investigated the influence of LY294002 on the autophagy with autophagy sensor LC3B-GFP. The result showed that the addition of 15 μM LY294002 into the RPE cells treated with 0.1 mM NAD^+^ and 300 μM H_2_O_2_ significantly decrease GFP-LC3B autophagic puncta compared with that in the RPE cells treated with 300 μM H_2_O_2_ and 0.1 mM NAD^+^ and that in the RPE cells treated with 15 μM LY294002 alone (Fig. 6B,D).

Then, we investigated the influence of LY294002 on the intracellular and intranuclear ROS level in the RPE cells treated with 300 μM H_2_O_2_, 0.1 mM NAD^+^. The result showed that the addition of 15 μM LY294002 into the RPE cells treated with 0.1 mM NAD^+^ and 300 μM H_2_O_2_ significantly increased intracellular and intranuclear ROS level compared with that in the RPE cells treated with 300 μM H_2_O_2_ and 0.1 mM NAD^+^ or that in the REP cells treated with 15 μM LY294002 alone (Fig. 6E–G). The results indicated that NAD^+^ decreased intracellular and intranuclear ROS level in H_2_O_2_-induced RPE might mediate through the up-regulation of autophagy. Furthermore, to determine the effect of inhibition of autophagy on the activation of PARP-1, we investigated the expression of PARP-1 by western blot. The result showed that addition of 15 μM LY294002 into the RPE cells treated with 0.1 mM NAD^+^ and 300 μM H_2_O_2_ significantly increased the expression of cleaved PARP-1 (active form of PARP-1) compared with that in the RPE cells treated with 300 μM H_2_O_2_ and 0.1 mM NAD^+^ or that in the REP cells treated with 15 μM LY294002 alone. No significant difference was observed in the expression of cleaved PARP-1 between the RPE cells treated with 0.1 mM NAD^+^ and 300 μM H_2_O_2_ and the REP cells treated with 15 μM LY294002 alone. Similarly, no significant difference was observed in the expression of PARP-1 among the three groups (Fig. 6H–J).

## Discussion

Clinically, treatment of AMD remains insufficient. NAD^+^ has been proposed to be a novel and inexpensive cytoprotective agent for its protection against cell death induced by oxidative stress. However, the effect of NAD^+^ on the oxidative stressed-RPE cell death is unknown. The questions are addressed in the present study.

### NAD^+^ improved the viability of RPE cell treat with H_2_O_2_

NAD^+^, a hydride-accepting and hydride-donating coenzyme, has central roles in cellular metabolism and energy production. Exogenous NAD^+^ supplement has been reported to protect cell from death induced by oxidative stress^17^,^23^. Besides, study on the light-induced retinal damage shows that NAD^+^ treatment attenuates photoreceptor degeneration and RPE cell death triggered by zinc toxicity, a result from pathologic light exposure^24^,^25^. All these studies suggest that NAD^+^ might be a novel cytoprotective agent against oxidative stress or zinc toxicity. In line with these studies, our results showed that 0.1 mM NAD^+^ administration to 300 μM H_2_O_2_-treated RPE cells significantly improved the cell viability (Fig. 1).

A number of studies have shown that oxidative stress or zinc toxicity can induce DNA damage and cell death by causing intracellular NAD^+^ depletion or ATP depletion^24^,^25^,^26^,^27^. In the present study, neither NAD^+^ depletion by measurement of intracellular NAD^+^ levels and NAD^+^/NADH ratios nor inhibition of NAD^+^ synthesis by measurement of gene expression of seven enzymes involved in NAD^+^ biosynthesis was detected in the RPE cells treated with 300 μM H_2_O_2_ (Fig. 2). *In vitro* study has shown that NAD^+^ treatment can prevents the decline of ATP levels^28^. Hence, it is possible that NAD^+^ treatment may improve H_2_O_2_-treated RPE cells viability by preventing ATP depletion.

### NAD^+^ protected RPE cell from necrotic death induced by H_2_O_2_

Oxidative stress is considered to play significant role in the pathogenesis of AMD. It triggers RPE cells progressive damage by accumulation of ROS which contributes to protein mis-folding and evoking functional abnormalities during RPE cellular senescence^29^. In the present study, we showed that NAD^+^ treatment significantly decreased not only intracellular ROS level but also intranuclear ROS level induced by H_2_O_2_ (Fig. 3). Therefore, it is possible that the protection of NAD^+^ on the RPE cell death may associate with its decline in intracellular ROS.

Previous studies implicate that apoptosis is a main mechanism for oxidative stress-induced RPE cell death^30^,^31^. However, recent study of the systematic analysis of RPE cell death induced by oxidative stress indicates that necrotic death is a major type of cell death in RPE cells in response to oxidative stress^13^. In the present study, annexin V/PI staining assay detected by HCS showed that the percentage of cells positive for PI staining significantly increased in the RPE cells treated with 300 μM H_2_O_2_ for 24 hours. Also, we investigated the expression of caspase-3, apoptosis-related marker, by the western blot assay. The results showed that no obvious alterations in the expressions of caspase-3 and cleaved caspases-3 were observed. Our result further supports the idea that necrotic cell death is a major death form for RPE in response to oxidative stress (Fig. 4).

Poly (ADP-ribose) polymerase-1 (PARP-1), chromatin-binding and transcription-related protein, reportedly mediates oxidative stress–induced cellular injury and necrotic cell death^32^. In the present study, we showed that the expression of cleaved PARP-1 significantly increased in RPE cells treated with 300 μM H_2_O_2_ compared with that in control RPE cells. No significant difference in the expression of PARP-1was observed between RPE cells without or with H_2_O_2_ treatment. The results suggest that oxidative stress induced the necrotic death of RPE cells mediated by activation of PARP-1.

To determine the protection of NAD^+^ against necrotic death of RPE cells, we investigated the effect of NAD^+^ on the activation of PARP-1 and necrotic death of RPE cells induced by oxidative stress. The results showed that 0.1 mM NAD^+^ significantly decreased the necrotic death of RPE cell induced by 300 μM H_2_O_2._ In addition, western bolt result showed that NAD^+^ significantly decreased the expression of cleaved PARP-1. The results suggested that exogenous NAD^+^ might block the oxidative stress-induced activation of PARP-1 and subsequently necrosis of RPE cells.

### Up-regulated autophagy by exogenous NAD^+^ protected RPEC cells from death induced by H_2_O_2_

Autophagy, a highly conserved cellular degradation pathway for the clearance of damaged or superfluous proteins and organelles, can be induced by various cellular factors and multiple cellular stresses^33^. Although accumulation of cellular oxidative stress and increased generation of ROS have been proposed to induce autophagy^34^,^35^,^36^, recent study shows that the increased oxidative stress/ROS does not result in stimulation of autophagy or mitophagy in cardiomyocytes^37^. In the present study, we also did not detect any induction of autophagy in the RPE cells treated with H_2_O_2_ through the assays of LC3B immunoblotting, LC3B-GFP puncta formation and autophagosome formation by TEM (Fig. 5). The results suggested that the increased oxidative stress/ROS might not be essential for the induction of autophagy in cultured RPE cells treated with H_2_O_2_. However, the increased ratio of LC3B-II/LC3B-I, increased GFP-LC3B positive cells and autophagosome structure were observed in cultured RPE cells treated with 0.1 mM NAD^+^ and 300 μM H_2_O_2_. The results suggested that exogenous NAD^+^ induced autophagy in the H_2_O_2_ treated-RPE cells. Mammalian target of rapamycin (mTOR) pathway has been known as a key regulator of autophagy^38^,^39^. Studies have been shown that SIRT1 positively regulates autophagy by inhibition of mTOR pathway^40^,^41^. However, in our study, no significant difference in the SIRT1 expression was observed in the RPE cells treated with 0.1 mM NAD^+^ and 300 μM H_2_O_2_ compared with that in the RPE cells treated with 300 μM H_2_O_2_ alone, or in the control RPE cells for 24 hours (data not shown). Study on cardiac hypertrophy shows that exogenous NAD^+^ elevates cellular NAD^+^ levels and activates SIRT3. SIRT3 activation stabilizes the activity of the LKB1-AMPK signaling pathway and blocks activity of mTOR^42^. Whether the induction of autophagy by exogenous NAD^+^ in the H_2_O_2_-treated RPE cells is through activation of SIRT3 and subsequently the inhibitory effect on mTOR pathway needs further investigated.

Autophagy is implicated to play pro-survival function in necrotic cell death^22^. A recent report shows that up-regulated autophagy protects cardiomyocytes from oxidative stress-induced toxicity^37^. Besides, autophagy has been proposed to serve to reduce ROS/oxidative stress level by removal of damaged mitochondria or oxidized proteins^43^,^44^. It is postulated that a breakdown in the recycling capacity of autophagy may be associated with accumulation of proteins and damaged organelles which are a general observation in the aging RPE as well as in AMD^45^. In line with those reports, the present study showed that treatment with LY294002, inhibitors of autophagy, resulted in significant decrease in cell viability in the RPE cell treated with 0.1 mM NAD^+^ and 300 μM H_2_O_2_. Meanwhile, the addition of 15 μM LY294002 into the RPE cells treated with 0.1 mM NAD^+^ and 300 μM H_2_O_2_ significantly decrease GFP-LC3B autophagic puncta. The results indicated that LY294002 inhibited the autophagy induced by NAD^+^ in the 300 μM H_2_O_2_ treated RPE cells. In addition, treatment with LY294002 also resulted significantly increased in ROS level/oxidative stress and the expression of cleaved PARP-1 level in the RPE cell treated with 0.1 mM NAD^+^ and 300 μM H_2_O_2_ (Fig. 6). Our results suggested that up-regulation of autophagy might be the major mechanism underlying the protection of NAD^+^ on the RPE cell against necrotic death induced by H_2_O_2_. However, whether the reduced ROS/oxidative stress level by up-regulated autophagy is through the removal of damaged mitochondria or oxidized proteins needs further investigated.

## Conclusion

**Figure 7:**
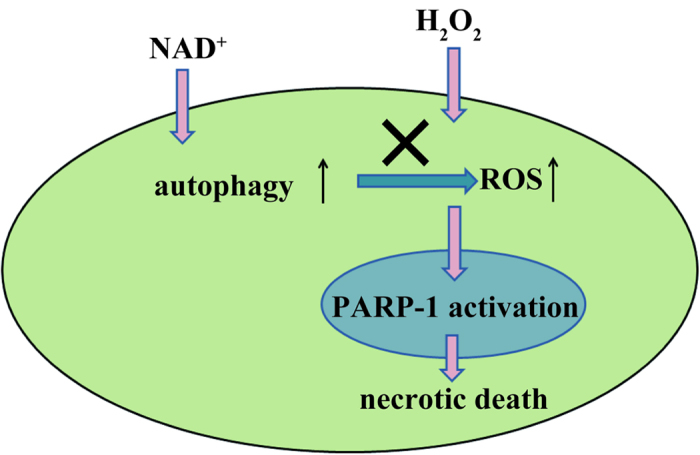
A schematic illustrates the protection of exogenous NAD^+^ administration against RPE cell death induced by H_2_O_2_. Exogenous NAD^+^ administration up-regulates the autophagy in H_2_O_2_-treated RPE cells. As the consequence, it decreases the intracellular ROS levels and inhibits the activation of PARP-1 and protects RPE cell from necrotic death.

In conclusion, this study has provided the first evidence that up-regulated autophagy by NAD^+^ reduced the oxidative stress in RPE cells. In turn, it protects RPE cells from PARP-1 mediated-necrotic cell death induced by oxidative stress (Fig. 7). The results suggest that exogenous NAD^+^ administration may be a novel, inexpensive, and effective treatment for preventing RPE cell death in AMD pathology. Further animal studies are required to explore this treatment value.

## Additional Information

**How to cite this article**: Zhu, Y. *et al.* Exogenous NAD^+^ decreases oxidative stress and protects H_2_O_2_-treated RPE cells against necrotic death through the up-regulation of autophagy. *Sci. Rep.* **6**, 26322; doi: 10.1038/srep26322 (2016).

## Supplementary Material

Supplementary Information
