# Supplementary material for: Exogenous NAD+ decreases oxidative stress and protects H2O2-treated RPE cells against necrotic death through the up-regulation of autophagy
Source: Sci Rep. 2016 May 31;6:26322. doi: 10.1038/srep26322 (PMC4886526; doi:10.1038/srep26322)
Supplement: Supplementary Information [file srep26322-s1.doc]

**Exogenous NAD^+^ decreases oxidative stress and protects H_2_O_2_-treated RPE cells against necrotic death through the up-regulation of autophagy**

Ying Zhu ^a1^, Ke-ke Zhao ^b1^, Yao Tong^a^, Ya-li Zhou^a^, Yi-xiao Wang^a^, Pei-quan Zhao^a^*, Zhao-yang Wang ^a^*

^a^ Department of Ophthalmology, Xinhua Hospital, Shanghai Jiaotong University Scool of Medicine, Shanghai, China.

^b^ Department of Ophthalmology, Shanghai Children’s Medical Center, Shanghai Jiaotong University School of Medicine, Shanghai, China.

1 These authors contributed equally to this work.

* These authors are the co-correspondence authors to this paper

**Correspondence:** Zhao-yang Wang MD, PhD. Department of Ophthalmology, Xinhua Hospital, Shanghai Jiaotong University School of Medicine, Shanghai, China. ([zhaokekewzy@hotmail.com](mailto:zhaokekewzy@hotmail.com))

**Correspondence:** Pei-quan Zhao MD. Department of Ophthalmology, Xinhua Hospital, Shanghai Jiaotong University School of Medicine, Shanghai, China. ([zhaopeiquan@126.com](mailto:zhaopeiquan@126.com))

**Table of contents**

**Supplementary Figures**

Supplementary Figure S1. Effect of NAD^+^ on the cell viability of RPE cells. page 3

Supplementary Figure S2. Autophagy in the cultured RPE cells detected by GFP-LC3 kit. page 4


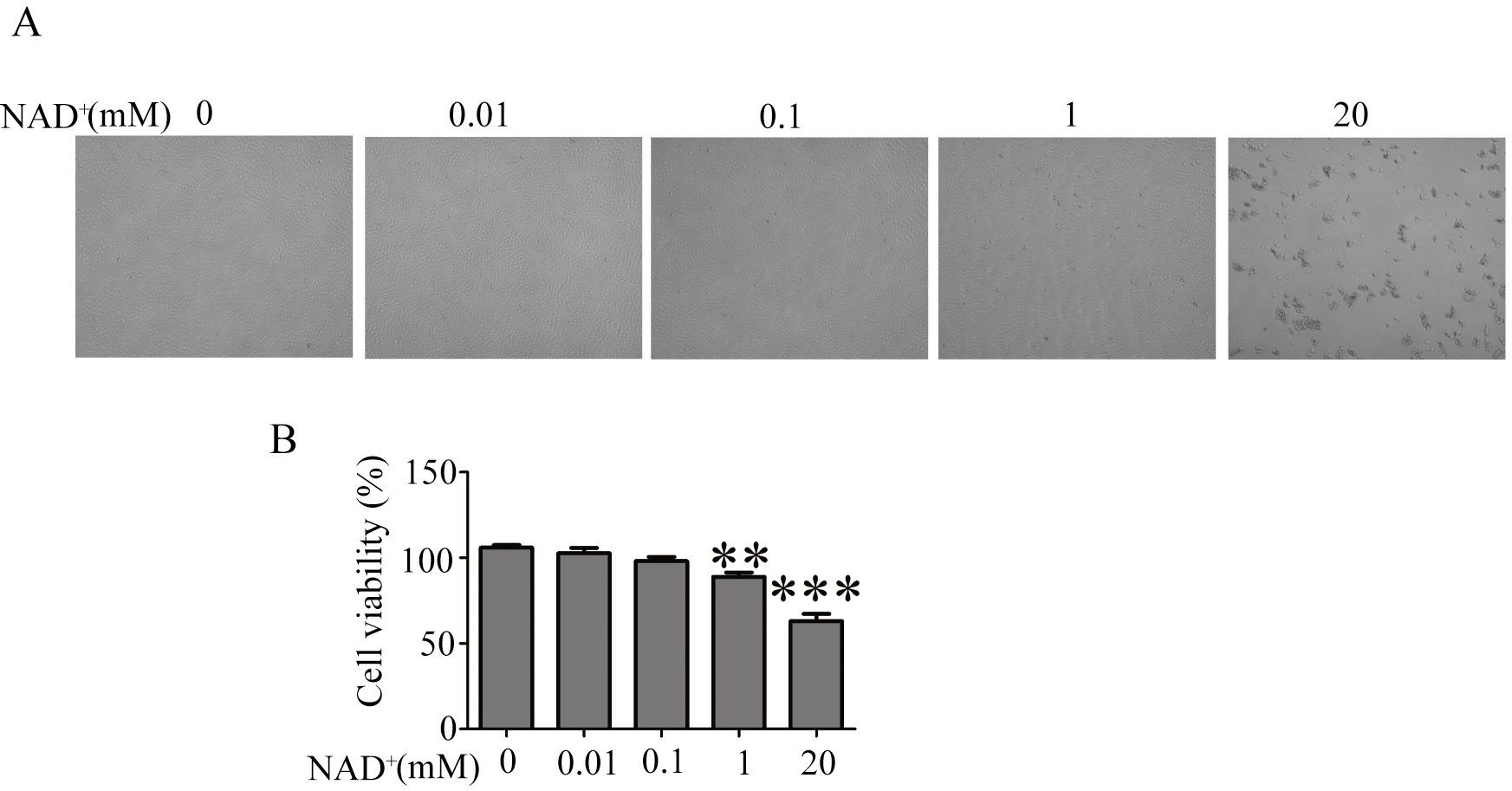


**Supplementary Figure S1.**  **Effect of NAD^+^ on the cell viability of RPE cells.** (A) Representative images show the damage of RPE cells treated without (0), or with different concentration of NAD^+^ (0.01 mM, 0.1 mM, 1 mM, 20 mM) for 24 hours. (B) Cell viability of RPE cells treated without (0), or with different concentration of NAD^+^ (0.01 mM, 0.1 mM, 1 mM, 20 mM) for 24 hours was assessed by CCK8. Data are expressed as mean±SEM from at least three independent experiments; **P<0.01, ***P<0.001 versus cell viability of RPE cells without any treatment.


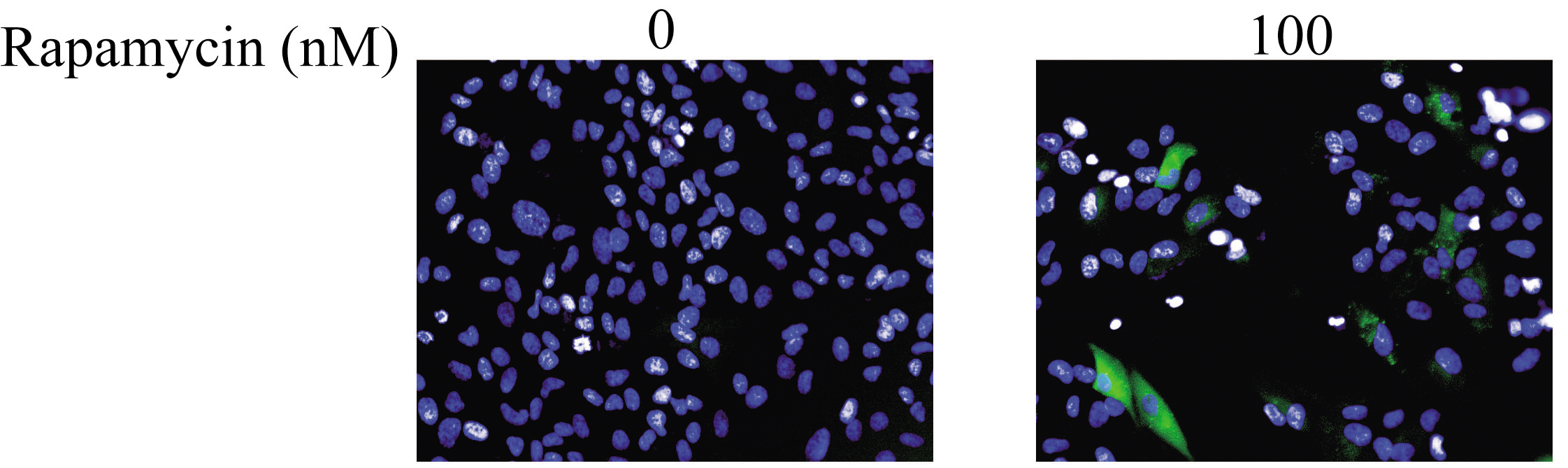


**Supplementary Figure S2.** **Autophagy in the cultured RPE cells detected by GFP-LC3 kit.** The cultured RPE cells were transfected with GFP-LC3 and then treated with or without 100 nM rapamyicin for 12 hours. The autophagy was examined with HCS. HCS image shows that autophagic puncta significantly increased in the cultured RPE cells treated with 100 nM rapamycin compared with that in the cultured RPE cells without any treatment.
